# 

**Published:** 1888-03-17

**Authors:** 


### CONTENTS.

**Registration: A Word to Nurses** - 405

**The Doctor's Pulpit**.—The Morals of the Workshop - 4[illegible]7

**The National Pension Fund** - 408

**Aids to Nursing.** By M. M. BASIL, M.A. M.B., Author of "The Commoner Accidents to Life and Limb"—Noise - 409

**The Nursing Mirror.**—The Art of Nursing as a Part of the Education of Women.—Winter Nursing Engagements.—Homes Abroad for Nurses.—Nursing in America - 410

**Scotch Physicians and Surgeons.** By C. G. FURLEY.—Sir Robert Christison - 411

**Notes and News** - 412

**Everybody's Page.**—How to Live to One Hundred.—A Word on Exercise.—A Welcome Reprieve, etc. - 414

**Annotations.**—The World and its Pedagogues.—An Experimental Test.—Can a Man of Science Wonder? - 415

**Round About the Asylums.** By a MEDICAL SUPERINTENDENT - 416

**Rachel Challice's Vow.** By LINA NEVILL, Author of "A Future on Trust," etc. Chapter xiv.—Found - 417

**The Editor's Letter-Box.**—Convalescent Home, Saltburn-by-the-Sea.—The Hospitals Association and its Seceding Off-shoot—The Late Dr. Anna Kingsford.—A Bequest to the Middlesex Hospital - 419

**The St. John's Hospital Scandal** - 419

**Amusements with Profit for all Ages.**—For Men and Women.—Children's Corner.—Puzzles, etc. - 420

